# The influence of age and surface compliance on changes in postural control and attention due to ankle neuromuscular fatigue

**DOI:** 10.1007/s00221-013-3795-7

**Published:** 2013-12-25

**Authors:** Etienne J. Bisson, Yves Lajoie, Martin Bilodeau

**Affiliations:** 1 Faculty of Health Sciences, School of Human Kinetics, University of Ottawa, 125 University Private, Ottawa, ON K1N 6N5 Canada; 2 Aging and Movement Research Laboratory, Élisabeth Bruyère Research Institute, 43 Bruyère Street, Ottawa, ON K1N 5C8 Canada; 3 Faculty of Health Sciences, School of Rehabilitation Sciences, University of Ottawa, 451 Smyth Road, Ottawa, ON K1H 8M5 Canada

**Keywords:** Muscle fatigue, Balance, Aging, Proprioception, Sensory integration, Dual-task

## Abstract

The reduction in the quality and integration of sensory information with aging could increase the alterations in postural control associated with muscle fatigue observed in younger adults. This study aimed to compare changes in postural control and attentional demands due to ankle muscle fatigue, with intact and reduced proprioceptive information at the ankle, between young and older adults. Eleven young (24 ± 4 years) and 13 older (65 ± 4 years) men stood quietly on a force platform (blindfolded) under four experimental conditions (combinations of firm (FS)/compliant (CS) surfaces and single/dual tasks), before and immediately after a fatiguing exercise. The fatiguing exercise, performed on a dynamometer, consisted of maintaining an isometric contraction of the plantarflexors at 50 % of maximum until exhaustion. Both COP sway area and COP sway velocity were greater on the CS compared to FS and increased with fatigue for both groups in all conditions. COP sway area showed a greater increase with fatigue in older adults when standing on the CS. Reaction time (secondary task) increased significantly after fatigue, but only for older adults when standing on the CS. The effects of fatigue on postural control are more important when proprioceptive information at the ankle is altered. In particular, older adults had more difficulty and may have needed more attention to stand quietly, compared with young adults.

## Introduction

Neuromuscular or muscle fatigue can alter postural control, with most studies showing an increase in sway amplitude or velocity in young adults (Paillard 2012). Numerous studies examining the effect of ankle muscle fatigue on postural control have suggested a link between an alteration in proprioception and an increase in sway with fatigue (Salavati et al. 2007; Gribble and Hertel 2004; Vuillerme et al. 2002a). According to the re-weighting hypothesis (Peterka 2002; Maurer et al. 2006), re-weighting sensory information from less or altered to more reliable sources would be required to maintain stability in such a condition (fatigue). In fact, studies have shown a modulation of the effect of fatigue on postural control according to available sources of sensory information (e.g., eyes closed vs. eyes open) (Boyas et al. 2011; Thedon et al. 2011; Gimmon et al. 2011; Bisson et al. 2010). Furthermore, when proprioceptive input is less reliable [e.g., on a compliant surface (CS)], postural control alterations due to ankle muscle fatigue can be exaggerated (Bisson et al. 2012) due to an additive effect: altered joint position sense (Allen et al. 2010) and force sense (Vuillerme and Boisgontier 2008) as a result of muscle fatigue and further alteration in kinesthesia caused by the CS (Horak and Hlavacka 2001).

The impact of muscle fatigue on postural control in older adults may be more pronounced than in young adults since their proprioceptive and neuromuscular systems are less efficient. Neuromuscular fatigue mechanisms can also be influenced by age, and this has been shown to be task dependent (Allman and Rice 2002). Interestingly, a number of studies have shown greater sway during quiet standing in older adults after fatigue (Moore et al. 2005; Egerton et al. 2009), but no direct comparison has been made with young adults. Furthermore, these studies were conducted on a firm and stable surface. It is known that age-related differences in postural control are particularly evidenced when inputs from two of the three sensory systems controlling posture are not available/reliable (e.g., vision blocked and proprioception altered). No study has examined the effect of fatigue on postural control in this condition in older adults. These individuals may present with greater fatigue-related alterations in sway with reduced proprioceptive information, because they have more difficulty re-weighting less-to-more reliable sensory information (Redfern et al. 2001; Hay et al. 1996). Also, as suggested by Egerton et al. (2009), even if postural control alterations due to muscle fatigue are similar between older and young adults, a greater increase in attentional demands after fatigue may be observed in the former group.

The goal of this study was to compare changes in postural control and attentional demands due to ankle muscle fatigue, on both a firm surface (FS) and a CS, between young and older healthy adults. Our main hypotheses were that (1) we would observe an increase in all sway variables with fatigue in both young and older adults, but this would be more pronounced in the latter group and on the CS (Bisson et al. 2012) and (2) we would document a decrease in performance of a secondary cognitive task with fatigue, which would also be more pronounced on the CS and for older adults.

## Methods

### Participants

Eleven young (24 ± 3 years, 181.9 ± 6.6 cm, 75.2 ± 9.8 kg) and 13 older men (65 ± 4 years, 175.3 ± 4.1 cm, 78.5 ± 9.6 kg) were recruited. None of the participants had a history of falls in the past year, and all reported being healthy. The older men scored between 28.5 and 30/30 (mean = 29.7) on the Mini Mental State Exam (Folstein et al. 1975). No participants had diminished protective plantar sensation when tested with the Semmes–Weinstein monofilaments (Feng et al. 2009). Both groups included participants with low (3 young, 3 older), moderate (3 young, 6 older) and high levels of physical activity (5 young, 4 older), as assessed with the Godin Leisure-Time Exercise Questionnaire (Godin and Shephard 1997). The study was approved by the University of Ottawa and the Bruyère Continuing Care Research Ethics Boards, and written informed consent was obtained prior to enrollment.

### Procedures

**Fig. 1:**
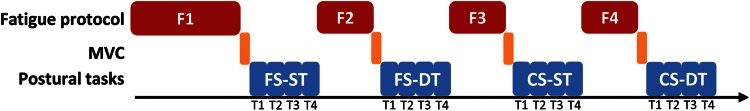
Schematic of the post-fatigue period. After baseline assessment of MVC and each postural tasks, participants performed the fatigue protocol four times (F1–F4), each followed with a MVC and four trials (T1–T4) of one postural task (task order counterbalanced between participants). *MVC* maximal isometric contraction, *FS* firm surface, *CS* compliant surface, *ST* single task and *DT* dual-task

Following sufficient practice, standing postural control and attentional demands in a dual-task paradigm were tested during two distinct groups of trials: (a) baseline trials and (b) post-fatigue trials. A schematic of the post-fatigue period is presented in Fig. 1. To minimize recovery during post-fatigue trials, the fatigue protocol was repeated between tested conditions (F1, F2, F3, F4).

Participants were first secured in a dynamometer chair. After a brief warm-up period, they performed three maximal isometric voluntary contractions (MVC) in plantarflexion, where the highest peak torque was considered the participant’s baseline MVC.

For the postural task, participants had to stand as still as possible with their feet together, their arms by their sides and blindfolded (opaque ski goggles) in four different conditions: quiet standing alone and while performing a secondary task (single vs. dual-task conditions), both on a FS and a CS. Each condition comprised four 30-s trials. The order of the four postural conditions was counterbalanced. For trials with the CS, participants were asked to stand on a block of dense foam placed on the force platform. For dual-task condition trials, participants had to prioritize the postural task while responding to a secondary task as best as they could. The secondary task consisted of a choice reaction time (CRT) task, where two different auditory stimuli were presented. Participants needed to respond “TIE” when the auditory cue was a high-pitch sound (3,000 Hz, 50 ms) and respond “TOE” when the auditory cue was a low-pitch sound (250 Hz, 50 ms). Six stimuli were presented per trial in a quasi-random manner with at least two stimuli of each sound given per trial. Four trials of seated CRT were also performed prior to the baseline trials and following the post-fatigue trials. While lip movement may increase postural sway during the dual task (Dault et al. 2003), the six verbal responses per trial took only a fraction of the 30-s trial used to measure each sway parameters. Thus, we are confident that lip movements had little to no effects on our postural data.

The fatigue protocol used in this study has been described in detail elsewhere (Bisson et al. 2012). A custom-built attachment to a BIODEX dynamometer (System III, Shirley, NY) was used to fatigue the plantarflexor muscles of both ankles simultaneously. The fatigue task consisted of a continuous isometric contraction of the plantarflexors at 50 % MVC until failure. The fatigue protocol was ended when participants were unable to hold 50 % MVC for five consecutive seconds. After completion of each bout of fatiguing exercise, participants were asked to perform a MVC before transferring to the force platform (2 m away) to perform the postural tasks.

### Data acquisition and statistical analyses

#### Fatigue characterization

Muscle fatigue was quantified by measuring the MVC torque after each fatigue protocol (F1, F2, F3, F4) and comparing this value to the baseline MVC. The time (s) elapsed from the start of the isometric contraction until failure was also recorded for each fatigue protocol (time to failure). A two-way mixed-model analysis of variance (ANOVA) was performed on the MVC torque and time to failure to analyze the effect of group (young, older) and fatigue protocol (MVC torque: baseline, F1, F2, F3, F4; time to failure: F1, F2, F3, F4).

#### Sway parameters

**Fig. 2:**
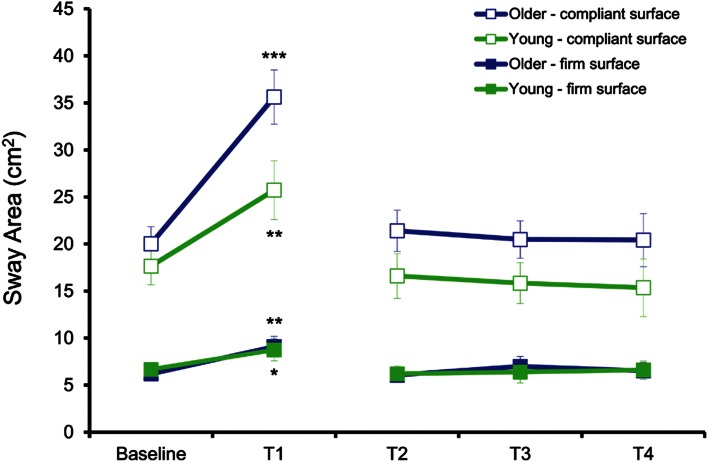
Mean and standard deviation of COP sway area during each condition for both groups. Statistical analysis between baseline and T1 showed that COP sway area was greater when standing on the CS compared to FS at all time points (*p* < 0.001). COP sway area significantly increased with fatigue (T1) for both young and older adults when standing on the FS and the CS (*p < 0.05, **p < 0.01, ***p < 0.001). COP sway area was significantly different (*p* < 0.05) between groups only at T1 when standing on the CS, suggesting a greater increase for the older adults during this condition. For clarity purposes, significance symbols are only shown to depict differences between baseline and T1

**Fig. 3:**
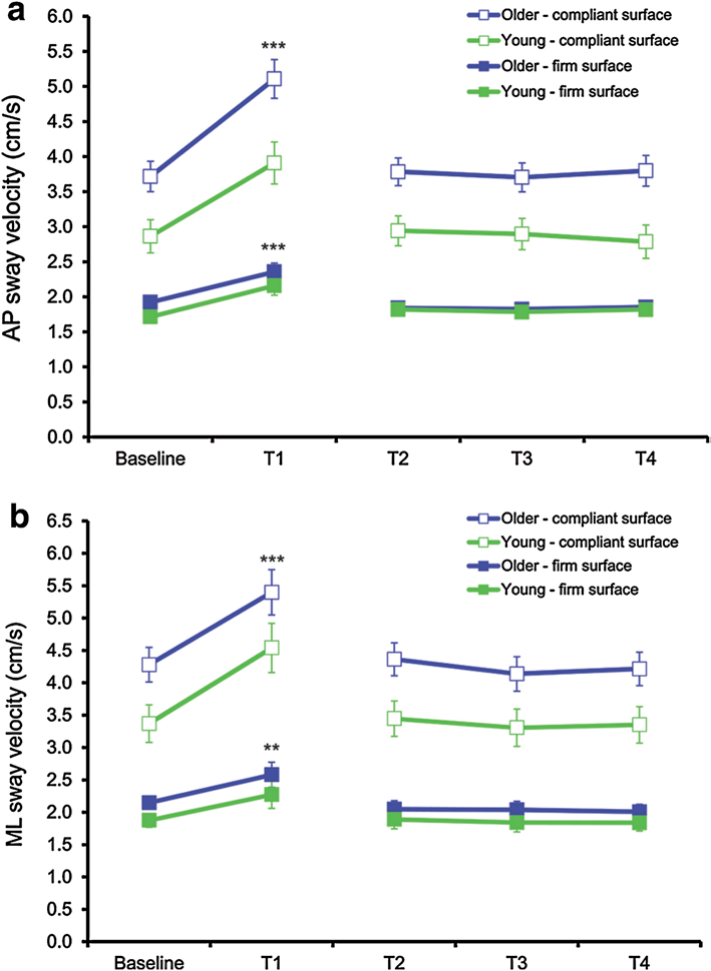
Mean and standard deviation of COP sway velocity in AP (**a**) and ML (**b**) during each condition for both groups. Statistical analysis between baseline and T1 showed that AP and ML COP sway velocity was greater when standing on the CS compared to FS at all time points (*p* < 0.001). AP and ML COP sway velocity significantly increased with fatigue (T1) when standing on the FS and the CS (**p < 0.01, ***p < 0.001). This increase was greater when standing on the CS (*p* < 0.001) but similar between groups. COP sway velocity was significantly different (*p* < 0.05) between groups on the CS (*p* < 0.05) but not on the FS. For clarity purposes, significance symbols are only shown to depict differences between baseline and T1

Center of pressure (COP) data were collected at a sampling rate of 50 Hz using an AMTI AccuGait force platform (Watertown, MA). COP sway during feet-together stance was characterized with three time-domain variables using BioAnalysis 2.1 software (Watertown, MA): the 95 % ellipse sway area, and medio-lateral (ML) and anterior–posterior (AP) COP sway velocity. For each condition, the mean of the four pre-fatigue trials (baseline) was calculated. In contrast, because the effect of muscle fatigue on COP sway has been shown to recover rapidly (Boyas et al. 2011; Harkins et al. 2005) and considering the present data which indicates that the effect of fatigue is apparent mainly in the first post-fatigue trial for all COP sway variables (see Figs. 2 and 3 in Results section), statistical analyses were performed only on this trial (T1) compared to the mean of the pre-fatigue trials (baseline). For each COP sway variable (area, AP and ML velocity), a four-way mixed-model ANOVA was used to analyze the effects of group (young, older), fatigue (baseline, T1), task (single, dual) and surface (FS, CS) on postural control.

#### Choice reaction time

The response time (referred to as CRT) was calculated as the time between the start of the auditory cue and the start of the vocal response. Both high-pitch (3,000 Hz, 50 ms) and low-pitch (250 Hz, 50 ms) auditory cues were generated by a computer and output through speakers placed at ear level. Verbal responses were recorded using a wireless voice recorder. The mean CRT (four trials) was calculated for both auditory cues (high and low-pitch cue) for the seated trials (before baseline trials and after post-fatigue trials), baseline trials and for each trial after fatigue (T1, T2, T3, T4). First, a three-way mixed-model ANOVA was performed on the seated CRT with the following factors: group (young, older), fatigue (baseline, post-fatigue) and type of cue (high-pitch, low-pitch) to confirm that muscle fatigue has no effects on verbal responses per se. Second, a four-way mixed-model ANOVA was performed on the standing CRT with the following factors: group (young, older), fatigue (baseline, T1), type of cue (high-pitch, low-pitch) and surface (FS, CS) to examine the effects of muscle fatigue on performance of a secondary task.

All statistical analyses were completed using PASW statistics 18 (IBM, Chicago, IL) with a significance level set at *p* < 0.05. Tests for sphericity were performed and values adjusted (using Greenhouse and Geisser adjustment) if found significant. Post hoc analyses were used when appropriate using a Bonferroni adjustment.

## Results

### Fatigue characterization

MVC torque showed significant main effects of group [*F*(1,21) = 4.73, *p* = 0.041], fatigue [*F*(4,84) = 172.7, *p* < 0.001] and a fatigue by group interaction [adjusted *F*(2.6,84) = 5.2, *p* = 0.005]. As presented in Table 1, MVC torque recorded after each fatiguing protocol was significantly different from baseline for both groups (*p* < 0.001). Young men had a greater MVC at baseline (*p* = 0.008), after fatigue protocol 1 (*p* = 0.027) and fatigue protocol 2 (*p* = 0.031) compared with older adults, but not after fatigue protocols 3 and 4.

**Table 1:** Fatigue characterization for young (*n* = 11) and older men (*n* = 13)

Baseline		Fatigue protocol			
		F1	F2	F3	F4
*MVC torque (N)*					
Young	266.1 (48.9)^a,b^	196.4 (39.0)^b^	197.1 (45.9)^b^	183.6 (41.8)	184.0 (39.4)
Older	219.1 (28.6)^a^	165.6 (25.4)	164.6 (22.6)	163.9 (27.3)	159.2 (25.6)
*Time to failure (s)*					
Young		126.1 (29.6)^c^	101.8 (25.9)	95.4 (22.0)	90.3 (18.3)
Older		176.2 (65.0)^c,d^	138.0 (49.6)^d^	123.6 (44.0)^d^	116.5 (25.7)^d^

*MVC* maximal voluntary contraction and *F* fatigue protocol

^a^MVC torque decreased for both groups from baseline to each fatigue protocol (*p* < 0.001)

^b^MVC torque was significantly greater for young compared with older adults (*p* < 0.05)

^c^Time to failure was longer for the first fatigue protocol (*p* < 0.001)

^d^Older adults had longer time to failure compared to young adults (*p* < 0.05)

Time to failure showed significant main effects of group [*F*(1,22) = 5.79, *p* = 0.025] and fatigue [*F*(3,66) = 31.0, *p* < 0.001]. As shown in Table 1, time to failure was significantly longer during the first bout of fatiguing exercise compared to all other bouts (*p* < 0.001). In addition, the time to failure of older men was longer compared with that of young men (*p* < 0.05).

### Effects of fatigue on COP sway parameters

For all COP parameters, the effect of the task (single vs. dual task) and all associated interactions were non-significant, suggesting that participants prioritized the primary (postural) task as instructed. Thus, for clarity purposes, COP data from both tasks were pooled and the subsequent results are presented as such. A summary of the statistical results are shown in Table 2.

**Table 2:** Statistical results of the comparison between groups on the effects of fatigue and types of surface for the COP sway parameters

Effects	COP sway parameters					
	Area		AP velocity		ML velocity	
	*F*(1,22)	*p*	*F*(1,22)	*p*	*F*(1,22)	*p*
Fatigue	56.24	<0.001	61.13	<0.001	24.35	<0.001
Surface	140.33	<0.001	186.81	<0.001	239.50	<0.001
Group		ns	4.44	0.047	8.29	0.009
Fatigue × surface	34.15	<0.001	35.47	<0.001	14.26	<0.001
Fatigue × group	4.62	0.047		ns		ns
Surface × group		ns	9.15	0.006	4.46	0.046
Fatigue × surface × group	4.42	0.043		ns		ns

*COP* center of pressure, *AP* antero-posterior, *ML* medio-lateral and *ns* not significant

COP sway area results (Fig. 2) showed (a) significantly greater COP sway area on the CS compared with the FS in both baseline and T1, and for both groups (*p* < 0.001); (b) a significant increase in COP sway area with fatigue for each surface and for both groups (*p* < 0.001) and (c) a significant difference in COP sway area between groups after fatigue (T1) when standing on the CS (*p* = 0.029) only. Thus, the increase in COP sway area due to fatigue was more pronounced for older men when standing on the CS (78 % increase) compared with when standing on the FS (46 % increase) and compared to their young counterparts when standing on either surfaces (CS: 46 % increase; FS: 31 % increase).

COP sway velocity results (Fig. 3) showed that AP and ML COP sway velocities were significantly greater during the CS condition compared with the FS condition at both baseline and post-fatigue and for both groups (*p* < 0.001). However, older men had greater COP sway velocity compared with young men on the CS (*p* < 0.05), but not the FS. Finally, ankle muscle fatigue significantly increased AP and ML COP sway velocity on both CS and FS, and for both groups (*p* < 0.01). This increase was more pronounced when standing on the CS (mean of both groups of 37 and 30 % increase for AP and ML, respectively) compared with when standing on a FS (mean of both groups of 24 and 21 % increase for AP and ML, respectively).

### Effects of fatigue on attention

Seated CRT showed no significant main effects of group [*F*(1,20) = 3.15, *p* = 0.091], fatigue [*F*(1,20) = 0.021, *p* = 0.886] or type of cue [*F*(1,20) = 0.793, *p* = 0.384], nor any interactions (*p* > 0.05). Both young and older adults were able to maintain their baseline seated CRT after the post-fatigue trials.

**Fig. 4:**
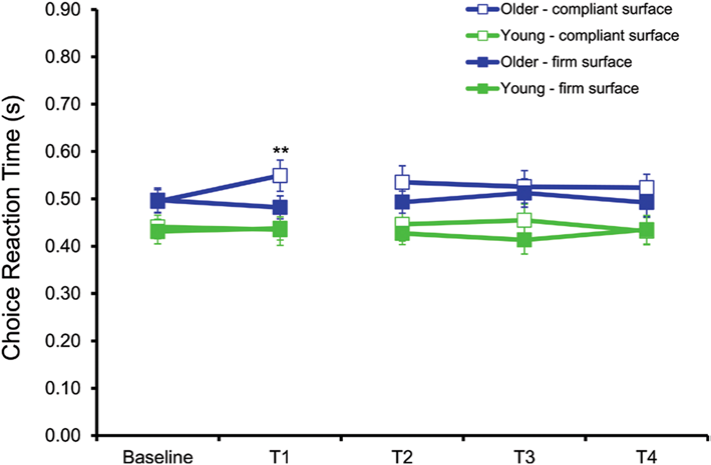
Mean and standard deviation for CRT during the dual-task conditions. Statistical analysis between baseline and T1 showed that CRT was significantly different between baseline and immediately after fatigue (T1) for the older adults when standing on the CS only (**p < 0.01)

Standing CRT showed significant main effects of type of cue [*F*(1,22] = 9.41, *p* = 0.006] and surface [*F*(1,22) = 4.73, *p* = 0.042]. Additionally, a significant fatigue by surface by group interaction [*F*(1,22) = 6.79, *p* = 0.017] was found. Participants responded faster to the low-pitch sound (mean CRT of 460 ms) compared to the high-pitch sound (mean CRT of 482 ms). As depicted in Fig. 4, pairwise comparisons showed that standing CRT increased significantly with fatigue in older men when standing on the CS (mean increase of 54 ms, *p* = 0.004) compared with when standing on a FS and compared with their young counterparts when standing on either surfaces (*p* > 0.05).

## Discussion

This study was the first to examine age-related differences in postural control alterations and possible increased attentional demands caused by ankle muscle fatigue when standing on a FS compared with a CS. All COP sway variables increased after fatigue. Both groups showed a greater increase in COP sway area with fatigue when standing on the CS compared with the FS. Older adults were also affected to a greater extent by muscle fatigue compared with young adults, but only when standing on a CS. Finally, only older men showed an increase in standing CRT with fatigue, and only when standing on the CS.

### Muscle fatigue effects on postural control in young adults

Localized muscle fatigue at the ankle has been shown to reduce joint position sense (Allen et al. 2010) and force sense (Vuillerme and Boisgontier 2008) through possible alterations in muscle spindles. Intensive work by Proske and colleagues (Allen et al. 2010; Tsay et al. 2012; Proske and Gandevia 2012) suggests that muscle fatigue induces a bias in the internal representation of the body (body schema). The consequences of this bias in postural control could be an increase in and/or improper corrective actions resulting in increased postural sway. In the present study, when the complexity of the task was fairly low (FS), young adults showed increased postural sway when fatigued. However, they were able limit postural control alterations due to fatigue possibly by re-weighting sensory inputs to more reliable sources such as vestibular and proprioceptive inputs from cutaneous mechanoreceptors (Paillard 2012).

Standing on an unstable surface alters the proprioceptive inputs from both the ankle joint and the foot (i.e., mechanoreceptors), increasing the difficulty to maintain stability. Our results showed increase in postural sway during such a task, but the increase was even greater after muscle fatigue. This finding is similar to others (Gimmon et al. 2011; Bisson et al. 2012) and suggests that postural sway increases in relation to the amount of reliable proprioceptive inputs available. Bias in body schema due to muscle fatigue, as suggested by Tsay et al. (2012), possibly accentuated the difficulty in maintaining stability during conditions where proprioceptive inputs were altered (when standing on a CS). The increased COP sway may also have been intentional to allow the use of sensory information from muscles, but with a higher threshold, following fatiguing activity.

Muscle fatigue could increase the central processing necessary for optimal postural control (Vuillerme et al. 2002b; Simoneau et al. 2006). Vuillerme et al. (2002b) and Simoneau et al. (2006) have shown changes in attentional demands with fatigue using a button-press reaction time task. However, using a voice reaction time task, our findings demonstrated that the attention allocated to the postural task did not increase with fatigue for young adults. This suggests that the attentional demands of postural control during muscle fatigue may be dependent on the secondary task performed. Furthermore, the fact that a CRT task (compared to a simple reaction time task) did not increase with fatigue suggests that this task specificity may not depend on the difficulty of the task, but more so on the type of response used (button press versus vocal response).

### Muscle fatigue effects on postural control of older adults

The effect of fatigue on COP sway of older adults reported here was greater compared with previous studies. For example, Egerton et al. (2009) have shown a modest effect of fatigue on COP sway displacement (5 % increase), whereas we showed a 46 % increase in COP sway area. This could be explained by the absence of vision, since visual information can attenuate the effect of muscle fatigue on postural control (Boyas et al. 2011; Bisson et al. 2010). However, as Egerton et al. (2009) failed to demonstrate, postural control in the present group of older adults was not more altered with fatigue than in young adults when standing on a FS. As for the young adults, possible bias in body schema may have resulted in more postural sway with fatigue. However, during a simple postural task such as standing with feet together, older adults, just like the young adults, were able to limit postural control alterations possibly by using other available proprioceptive inputs (e.g., mechanoreceptors at the foot).

With similar results, Egerton et al. (2009) suggested that the attention allocated to postural control could have increased with fatigue, making older adults at greater risk of falls. However, as observed here for young adults, older adults did not require more attention with fatigue on the FS, which suggests that for this condition, older adults were able to limit postural control alterations with a similar amount of attention as their younger counterparts.

Postural tasks where two sensory systems are altered (e.g., with eyes closed and standing on a CS) are more sensitive to differences between older and young adults (Woollacott et al. 1986). This is reflected in our results where no differences between groups were found in COP sway velocity when standing on a FS, but a greater velocity was observed when standing on the CS for older compared with young adults. However, the novelty of our work was to examine how older adults, compared to younger adults, can maintain stability with sub-optimal sensory information, beyond environmental factors (such as unstable surface). Further to previous studies that found greater postural control alterations due to muscle fatigue when the eyes are closed and the surface is unstable (Gimmon et al. 2011; Bisson et al. 2012), our findings are the first to show that these alterations are even greater in older adults. During muscle fatigue, the increase in COP sway velocity was similar across groups, which may suggest a similar increase in corrective actions between groups. COP sway area was similar between older (20.01 ± 1.83 cm) and young (17.64 ± 1.99 cm) adults before fatigue when standing on a CS, suggesting that the older group was highly functional. Although COP sway area increased for both groups after fatigue on the CS compared to the FS, older adults showed a greater increase (78 versus 46 % for young adults). Thus, with either reduced proprioception at the ankle and foot (CS) or when ankle muscles are fatigued on a FS, highly functional older adults are able to limit postural control alterations as well as young adults. However, when both conditions (muscle fatigue and standing on the CS) are combined, older adults have more difficulty in controlling their stability than young adults. Therefore, it seems that older adults have greater difficulty in maintaining their stability compared with young adults when task complexity is high and/or there is significant alteration in sensory inputs. By manipulating the sensory inputs (i.e., sensory organization test), studies (Whipple et al. 1993; Mujdeci et al. 2012; Pedalini et al. 2009; Cohen et al. 1996) have shown greater differences in postural sway in older adults compared to young adults during the most complex conditions (i.e., standing on a CS with sway-referenced visual information). Furthermore, greater instability during those conditions has been shown in older adults with a history of falls (Mujdeci et al. 2012; Wallmann 2001; Whitney et al. 2006). We could hypothesize similar results when older adults are standing on a CS with muscle fatigue.

Furthermore, with proprioceptive inputs potentially perturbed by two different sources (CS and muscle fatigue), older adults may have more difficulty re-weighting sensory information and recalibrating their body schema (Sturnieks et al. 2008). Based on the theory of multiple internal models (Wolpert and Kawato 1998), Boisgontier et al. (2013) recently showed that older adults were predominantly using an intermittent model of control during a position sense task. These authors showed that older adults were using sub-movements to control their movement trajectory instead of using a re-weighting process (continuous model of action), which led to greater errors during a more challenging task (time constraint). In relation to our results, using sub-movements instead of a re-weighting process may explain why older adults had greater sway area during the most challenging task. This could also lead to a greater increase in attentional demands with fatigue in this group compared with young adults (Lacour et al. 2008). Our results for the secondary task support this. When standing on a CS with eyes closed, older adults increased their CRT by 50 ms after muscle fatigue, whereas young adults showed no increase. These results contrast with the FS results, where peripheral inputs at the foot and ankle were still available to compensate for muscle fatigue. This reflects the importance of proprioceptive inputs for postural control and that older adults could be less efficient and may need more attention in using other sensory inputs to maintain stability during a complex postural task when their ankle muscles are fatigued.

It has to be noted that our findings can only be generalized to a highly active/functional older adult population. Although this can be considered a limitation of the present study, the effect of fatigue could even be greater with a more frail population (e.g., sedentary older adults, diabetics) for which the relative task complexity and sensory threshold increase. This warrants further investigation. Similar to most previous studies, we document the effect of muscle fatigue on conventional COP sway parameters, without reporting changes in complementary measures such as muscle activation, joint stiffness and proprioception. The interpretation of the findings could have been more complete with these measures, and future studies should focus on examining the link between postural variables and these complementary variables to better understand the compensatory strategies elicited in response to muscle fatigue.

## Conclusion

This study has shown greater postural control alterations due to ankle muscle fatigue when proprioceptive information at the ankle is less reliable (CS). Furthermore, older adults had more difficulty and may have needed more attention to stand quietly when fatigued, compared with young adults, but only in the context of standing on a CS without vision. To our knowledge, this study is the first to show fatigue-related differences between older and young adults on postural control in quiet standing. Considering an increase of 78 % in COP sway area and an increase of 50 ms in CRT, older adults may be temporarily at risk of a fall when fatigued and proprioceptive feedback is less reliable.

## References

[CR1] Allen TJ, Leung M, Proske U (2010). The effect of fatigue from exercise on human limb position sense. J Physiol.

[CR2] Allman BL, Rice CL (2002). Neuromuscular fatigue and aging: central and peripheral factors. Muscle Nerve.

[CR3] Bisson EJ, Chopra S, Azzi E, Morgan A, Bilodeau M (2010). Acute effects of fatigue of the plantarflexor muscles on different postural tasks. Gait Posture.

[CR4] Bisson E, Remaud A, Boyas S, Lajoie Y, Bilodeau M (2012). Effects of fatiguing isometric and isokinetic ankle exercises on postural control while standing on firm and compliant surfaces. J Neuroeng Rehabil.

[CR5] Boisgontier MP, Beets IA, Duysens J, Nieuwboer A, Krampe RT, Swinnen SP (2013). Age-related differences in attentional cost associated with postural dual tasks: increased recruitment of generic cognitive resources in older adults. Neurosci Biobehav Rev.

[CR6] Boyas S, Remaud A, Bisson EJ, Cadieux S, Morel B, Bilodeau M (2011). Impairment in postural control is greater when ankle plantarflexors and dorsiflexors are fatigued simultaneously than when fatigued separately. Gait Posture.

[CR7] Cohen H, Heaton LG, Congdon SL, Jenkins HA (1996). Changes in sensory organization test scores with age. Age Ageing.

[CR8] Dault MC, Yardley L, Frank JS (2003). Does articulation contribute to modifications of postural control during dual-task paradigms?. Brain Res Cogn Brain Res.

[CR9] Egerton T, Brauer SG, Cresswell AG (2009). Fatigue after physical activity in healthy and balance-impaired elderly. J Aging Phys Act.

[CR10] Feng Y, Schlosser FJ, Sumpio BE (2009). The Semmes Weinstein monofilament examination is a significant predictor of the risk of foot ulceration and amputation in patients with diabetes mellitus. J Vasc Surg.

[CR11] Folstein MF, Folstein SE, McHugh PR (1975). Mini-mental state. A practical method for grading the cognitive state of patients for the clinician. J Psychiatr Res.

[CR12] Gimmon Y, Riemer R, Oddsson L, Melzer I (2011). The effect of plantar flexor muscle fatigue on postural control. J Electromyogr Kinesiol.

[CR13] Godin G, Shephard R (1997). Godin Leisure-Time Exercise Questionnaire. Med Sci Sports Exerc.

[CR14] Gribble PA, Hertel J (2004). Effect of hip and ankle muscle fatigue on unipedal postural control. J Electromyogr Kinesiol.

[CR15] Harkins KM, Mattacola CG, Uhl TL, Malone TR, McCrory JL (2005). Effects of 2 ankle fatigue models on the duration of postural stability dysfunction. J Athl Train.

[CR16] Hay L, Bard C, Fleury M, Teasdale N (1996). Availability of visual and proprioceptive afferent messages and postural control in elderly adults. Exp Brain Res.

[CR17] Horak FB, Hlavacka F (2001). Somatosensory loss increases vestibulospinal sensitivity. J Neurophysiol.

[CR18] Lacour M, Bernard-Demanze L, Dumitrescu M (2008). Posture control, aging, and attention resources: models and posture-analysis methods. Neurophysiol Clin.

[CR19] Maurer C, Mergner T, Peterka RJ (2006). Multisensory control of human upright stance. Exp Brain Res.

[CR20] Moore JB, Korff T, Kinzey SJ (2005). Acute effects of a single bout of resistance exercise on postural control in elderly persons. Percept Mot Skills.

[CR21] Mujdeci B, Aksoy S, Atas A (2012). Evaluation of balance in fallers and non-fallers elderly. Braz J Otorhinolaryngol.

[CR22] Paillard T (2012). Effects of general and local fatigue on postural control: a review. Neurosci Biobehav Rev.

[CR23] Pedalini ME, Cruz OL, Bittar RS, Lorenzi MC, Grasel SS (2009). Sensory organization test in elderly patients with and without vestibular dysfunction. Acta Otolaryngol.

[CR24] Peterka RJ (2002). Sensorimotor integration in human postural control. J Neurophysiol.

[CR25] Proske U, Gandevia SC (2012). The proprioceptive senses: their roles in signaling body shape, body position and movement, and muscle force. Physiol Rev.

[CR26] Redfern MS, Jennings JR, Martin C, Furman JM (2001). Attention influences sensory integration for postural control in older adults. Gait Posture.

[CR27] Salavati M, Moghadam M, Ebrahimi I, Arab AM (2007). Changes in postural stability with fatigue of lower extremity frontal and sagittal plane movers. Gait Posture.

[CR28] Simoneau M, Begin F, Teasdale N (2006). The effects of moderate fatigue on dynamic balance control and attentional demands. J Neuroeng Rehabil.

[CR29] Sturnieks DL, St George R, Lord SR (2008). Balance disorders in the elderly. Neurophysiol Clin.

[CR30] Thedon T, Mandrick K, Foissac M, Mottet D, Perrey S (2011). Degraded postural performance after muscle fatigue can be compensated by skin stimulation. Gait Posture.

[CR31] Tsay A, Allen TJ, Leung M, Proske U (2012). The fall in force after exercise disturbs position sense at the human forearm. Exp Brain Res.

[CR32] Vuillerme N, Boisgontier M (2008). Muscle fatigue degrades force sense at the ankle joint. Gait Posture.

[CR33] Vuillerme N, Danion F, Forestier N, Nougier V (2002). Postural sway under muscle vibration and muscle fatigue in humans. Neurosci Lett.

[CR34] Vuillerme N, Forestier N, Nougier V (2002). Attentional demands and postural sway: the effect of the calf muscles fatigue. Med Sci Sports Exerc.

[CR35] Wallmann HW (2001). Comparison of elderly nonfallers and fallers on performance measures of functional reach, sensory organization, and limits of stability. J Gerontol A Biol Sci Med Sci.

[CR36] Whipple R, Wolfson L, Derby C, Singh D, Tobin J (1993). Altered sensory function and balance in older persons. J Gerontol.

[CR37] Whitney SL, Marchetti GF, Schade AI (2006). The relationship between falls history and computerized dynamic posturography in persons with balance and vestibular disorders. Arch Phys Med Rehabil.

[CR38] Wolpert DM, Kawato M (1998). Multiple paired forward and inverse models for motor control. Neural Netw.

[CR39] Woollacott MH, Shumway-Cook A, Nashner LM (1986). Aging and posture control: changes in sensory organization and muscular coordination. Int J Aging Hum Dev.

